# Identification of conserved genes involved in nitrogen metabolic activities in wheat

**DOI:** 10.7717/peerj.7281

**Published:** 2019-07-09

**Authors:** Lei Li, Hao Gong, Zhengxi Sun, Tao Li

**Affiliations:** 1 Jiangsu Key Laboratory of Crop Genetics and Physiology/Key Laboratory of Plant Functional Genomics of the Ministry of Education/Jiangsu Key Laboratory of Crop Genomics and Molecular Breeding, Yangzhou University, Yangzhou, China; 2 Jiangsu Co-Innovation Center for Modern Production Technology of Grain Crops, Yangzhou University, Yangzhou, China

**Keywords:** Wheat, Lesion mimic mutant, Nitrogen metabolism-related gene

## Abstract

Nitrogen (N) plays a very important role in crop growth and development. Many N-metabolism-related genes responsive to N application have been identified in many plants such as Arabidopsis, rice and maize; however, few genes have been reported in wheat, which is one of the most widely grown crops in the world. In this study, a wheat wild type with N dependent lesion mimic (LM) and its mutants without LM were used to identify conserved N-metabolism-related genes. *TaPAP*, *TaUPS* and *TaNMR* were differentially expressed among N levels both in the wild type and two of its mutants, and the expression patterns of these genes were further studied under application of three chemotypes of N (NH_4^+^_, NO_3^-^_ and NH_4_NO_3_). The results showed that these genes are conserved N-metabolism-related genes and *TaNMR* is a novel player in N-metabolism.

## Introduction

Wheat is one of the most widely grown crop species in the world, with an annual production about 749 billion kilograms. However, global wheat production needs to increase at least 1.8 times by 2050 to meet the challenge of food demands (http://faostat3.fao.org (2016)). Wheat productivity is heavily dependent on application of nitrogen (N) fertilizers. N is an essential element of amino acid (AA) and an essential macronutrient for growth and development in crop life cycle (Liu et al., 2015). Many different forms of N such as nitrate, ammonium, organic-N and other N-containing substances can be absorbed and assimilated by plants. Nitrate and ammonium are the two prominent forms of N taken up by many plants (Von Wittgenstein et al., 2014), although more than 90% of soil N is in the organic form (Li, Wang & Stewart, 2013).

In plants, two groups of ion transporter proteins, nitrate transporters (NRTs) and ammonium transporters (AMTs), are responsible for the uptake and transport of nitrate and ammonium, respectively (Couturier et al., 2007). Chlorate resistant 1 (CHL1), the first NRT1 transporter isolated (Tsay et al., 1993), belongs to NRT1/PTR (nitrate/peptide transporters) family responsible for the transportation of dipeptides in animals, fungi and bacteria (Wang, Hsu & Tsay, 2012). The second type of nitrate transporter NAR2 (nitrate transporter accessory protein), belonging to high-affinity nitrate transport system, was characterized in Chlamydomonas (Quesada, Galvan & Fernandez, 1994), Arabidopsis and rice (Feng et al., 2011). The third type of nitrate transporters is CLC (chloride-channel) family protein, which acts as anion channels or anion–proton exchangers (Barbier-Brygoo et al., 2011; Wang, Hsu & Tsay, 2012). AMTs comprise two discrete types, AMT1 and AMT2. AMT1 was identified as a high-affinity NH_4^+^_ transporter from yeast (Marini et al., 1994) and Arabidopsis (Ninnemann, Jauniaux & Frommer, 1994). An AMT2 variant, *AMT2;1*, was isolated from *Arabidopsis thaliana*, whereas the other variant AMT2s (referred to as MEP*α*) forms a sister clan to some fungal proteins from leotiomyceta, and several horizontal gene transfer events have been reported in the MEP family (McDonald, Dietrich & Lutzoni, 2011; Von Wittgenstein et al., 2014).

After the uptake of nitrate, it is first reduced by cytosolic nitrate reductase (NR) to nitrite, which is then imported into the chloroplast and reduced further by nitrite reductase (NiR) into ammonium. The latter is subsequently assimilated through the glutamine synthetase (GS) and glutamine-2-oxoglutarate aminotransferase (GOGAT), producing glutamate. In higher plants, two types of GOGAT, the ferredoxin-glutamine oxoglutarate aminotransferase (Fd-GOGAT) and the NADH-glutamine oxoglutarate aminotransferase (NADH-GOGAT) are participating in the synthesis of glutamate. The synthesis of glutamate is the starting point for the synthesis of most amino acids (Konishi et al., 2014; Krapp, 2015).

Although each amino acid has its unique catabolic pathway, all enzymes and metabolites involved in amino acid catabolism have the common characteristics across organisms. These metabolites of amino acid include ammonia, CO_2_, glucose, long-chain and short-chain fatty acids, H_2_S, ketone bodies, nitric oxide (NO), urea, uric acid, polyamines and other nitrogenous substances, which were generated under the catalyzing of enzymes such as glutamate dehydrogenase, branched-chain amino acid transaminase, phosphate-activated glutaminase, ornithine decarboxylase, NO synthase, lysine: *α*-ketoglutarate reductase, threonine dehydrogenase, arginase, cysteine dioxygenase, hydroxymethyltransferase, S-adenosylmethionine synthase, proline oxidase, glutamine:fructose-6-phosphate transaminase, D-amino acid oxidase, serine dehydratase, and glycine synthase (Wu, 2009). Previous studies showed that nitrogen metabolism is a complicated process associated with many aspects of the plant performance.

In wheat, the regulation of NRTs by N-status indicated their involvement in transportation of N-metabolism related substrates (Buchner & Hawkesford, 2014). The NAC (nitrate-inducible and cereal-specific NAM, ATAF, and CUC) transcription factor is involved in nitrate sensing and signaling. Overexpression of *TaNAC2-5A* in wheat enhanced root growth and nitrate influx rate, hence, increased its ability to acquire N (He et al., 2015). Nitrate N is reduced into ammonium N under the catalysis of NR and NiR. N starvation in wheat caused a significant decrease both in transcript levels and in NR and NiR activities (Balotf, Kavoosi & Kholdebarin, 2016).

We first reported a trait N dosage dependent hypersensitive reaction-like (HRL, also referred to as LM) in wheat variety P7001 (Li et al., 2016a). Our previous studies showed that wheat variety Ning7840 has LM phenotype at heading stage (Li & Bai, 2009; Li, Bai & Gu, 2012), while two EMS-induced mutants of Ning7840, *Dlm1* and *Dlm2*, showed the absence of LM (Li et al., 2016b). Interestingly, we found that Ning7840 and its two mutants mentioned above have markedly different responses to N application, where LM in Ning7840 was sensitive to N dosage whereas the absence of LM in the two mutants were insensitive to N dosage. Therefore, Ning7840 and its two mutants were used in the current study to screen conserved N-metabolism-related genes, as well as to analyze their expression patterns under the application of different N-chemotypes, which could be helpful for the understanding of molecular mechanisms underlying N-metabolism in wheat.

## Material and Methods

### Plant materials

Ning7840 (wild type, WT) is a spring wheat variety and has the LM trait under low N supply. *Dlm1* and *Dlm2* are EMS (ethyl methane sulfonate)-induced mutants with LM deficiency phenotype (Li et al., 2016b). These three lines were planted in plastic pots filled with 3 kg of soil. The N availability in the soil was assayed by the distillation method (Bremner, 1965). Plants were treated with three levels (0 g, 2 g and 4 g per pot) of N fertilizer (carbamide) at heading stage, where 1 g carbamide per pot roughly equals to 350 kg of N per hectare. The experiment was arranged in a randomized-complete-block design with two replicates (pots) and ten plants per replicate (pot). After being vernalized at 4 °C for four weeks, all plants were transplanted into a greenhouse at a day/night temperature of 22  ± 5 °C/17  ± 2 °C with supplemental light for 12 h in Yangzhou University, Yangzhou, Jiangsu province, China.

### Transcriptome analysis by RNA-sequencing

One week after carbamide treatment, total RNA of flag leaves of Ning7840, *Dlm1* and *Dlm2* were extracted (TRIzol reagent; Invitrogen) from three plants per line at heading stage. DNase (Invitrogen) and RNA easy Mini Kit (Qiagen) were used for removal of DNA and purification of RNA, respectively. An Agilent 2100 Bioanalyzer (Santa Clara, CA, USA) was used for detecting RIN (RNA integrity number) values (>8.0). The cDNA libraries for Ning7840, *Dlm1* and *Dlm2* were constructed and sequenced by the Biomarker Biotechnology Corporation (Beijing, China) (Lou et al., 2014; Wang et al., 2015). An Illumina HiSeq 2000 was used for next-generation sequencing. Transcriptome assembly, functional annotation and classification of unigene sequences followed Li et al. (2016b).

### Analysis of differentially expressed genes

The term “transcript” was used to refer to an integration of the three homeologous forms from the A, B and D genomes of hexaploid wheat, and they are assumed to have analogous functions, considering that they are sharing similarities of 90–99% at the nucleotide sequence and even higher at the amino acid sequence (Pellny et al., 2012). To evaluate the depth of coverage, the SOAPaligner software was used for realignment of all high-quality reads and the calculation of FPKM values (fragments per kb per million reads) (Mortazavi et al., 2008). After that, the expression level of uni-transcripts was calculated according to the FPKM values, and the false discovery rate (FDR) control method was used to calculate the threshold of *P* value in multiple tests. Only uni-transcripts with an absolute value of log2 ratio ≥2 and an FDR significance score <0.001 were used for subsequent analysis.

### Expression patterns of nitrogen metabolism-related genes

Ning7840 was planted in distilled water until three-leaf stage. The plants were then transferred to liquid MS medium (pH = 6) supplied with three N chemotypes (5 mMol ammonium succinate, 10 mMol potassium nitrate, 5 mMol ammonium nitrate), and 0 g of N treatment was used as a control check. An air pump was used for supplying oxygen. The experiment was arranged in three replicates (pots) and eight plants per replicate (pot). Total RNA from leaves were extracted at 0 h (h), 2 h, 6 h, 12 h, 1 day (d), 3 d and 7 d after N treatments. The procedure of RNA extraction was performed as described above. The oligo (dT) primer was used for the synthesis of first strand cDNA. The primer pairs for qPCR were designed based on the RNA-sequence from RNA-seq using Primer Premier 5. In the qPCR experiments, the wheat actin gene (Crismani et al., 2006) and three N-metabolism-related genes of interest were amplified with gene-specific primers (Table S1A). qPCR was performed using the SYBR PrimeScript RT-PCR Kit (TaKaRa, Japan). The threshold cycle (Ct) was determined by the default parameter of the Applied Biosystem 7900HT Real-Time PCR System. For each sample, three biological replicates and three experimental replicates were prepared and the experimental errors were calculated from the biological replications.

## Results

### The lesion mimic phenotype in Ning7840 is regulated by N

**Figure 1:**
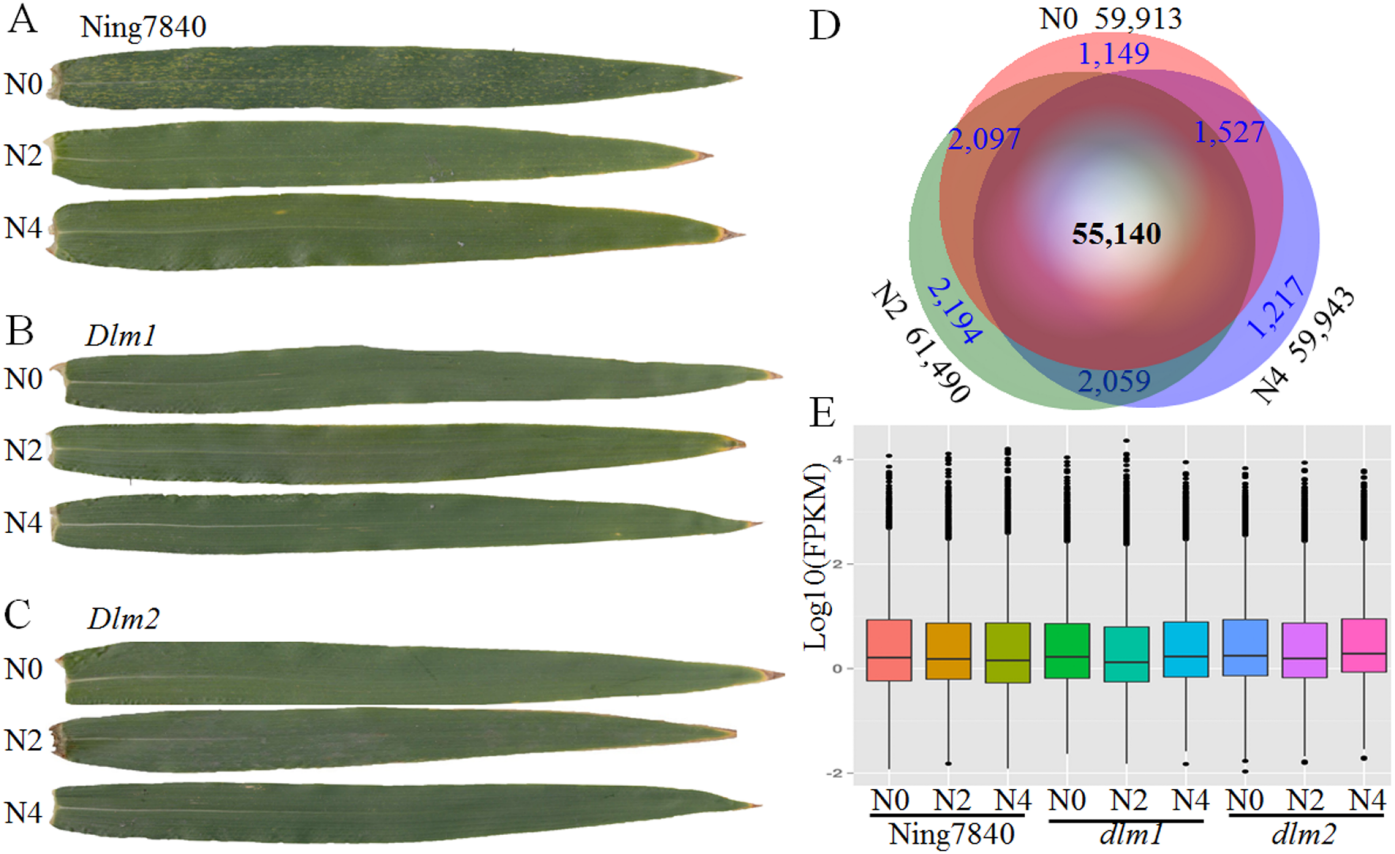
RNA-seq data and expressed transcripts under the different nitrogen treatments. N0, N2 and N4 represent the treatment with 0 g, 2 g or 4 g of carbamide per pot, respectively. (A) In Ning7840, small yellowish spots appear randomly on the leaves at heading stage under N0, whereas the spots are not observed under N2 and N4 treatments, and the yellowish spots did not appear on the leaves of *Dlm1* (B) and *Dlm2* (C) mutants, irrespective of N fertilizer dosage. (D) Venn diagram of transcripts unique and common across the three levels of nitrogen treatments and the number of expressed transcripts. (E) Distribution of FPKM in the nine libraries for the three genotypes under three N levels.

In the absence of pathogen, small yellowish spots appeared randomly on both sides of green leaves of Ning7840 at the heading stage in the control, whereas the spots were not observed in Ning7840 in the treatments of 2 g and 4 g carbamide per pot, and the N availability in the soil was 151 ± 5.2 mg/kg. For *Dlm1* and *Dlm2* mutants, the yellowish spots did not appear on the leaves irrespective of N dosages (Fig. 1A). These results suggest that the LM phenotype in Ning7840 is N dependent and high dosage of N suppresses the expression of LM trait.

### RNA sequencing data

After trimming ambiguous reads, low-quality reads, and adaptor sequences, a total of 350.31 million paired-end reads were generated from nine libraries, accounting for 70.76 Gb of clean data. The Q30 percentage (the percentage of bases in the reads with a phred quality equal or larger than 30) of the clean reads were higher than 86.3%, suggesting that the sequencing results are valid (Table S1B). A total of 65,383 transcripts were obtained from the nine assembled samples. The N50 (the length for which the sum of bases in the long contig of that length or longer is at least half the bases in the assembly) of transcripts is 1,425 nt. The size distribution of these transcripts is shown in Fig. S1.

Comparative analysis of the transcriptome revealed that across the three wheat lines (Ning7840, *Dlm1* and *Dlm2*) 57,468, 60,445 and 59,333 transcripts were found under the treatments with 0 g, 2 g and 4 g of carbamide per pot, respectively. In total, 55,140 genes were commonly expressed over the treatments (Fig. 1B), and many of them were quantitatively regulated under different dosages of carbamide (Table S2). The average expression levels of genes under N0, N2 and N4 treatments are 12.22, 12.54 and 12.56 FPKM, respectively. Expression levels of most genes are low (FPKM < 10) to moderate (between 10 and 100). A small fraction (1.91–2.04%) were expressed at extremely high levels (FPKM > 100) (Fig. 1C).

### Differentially expressed genes (DEGs) and conserved N-metabolism-related genes

**Figure 2:**
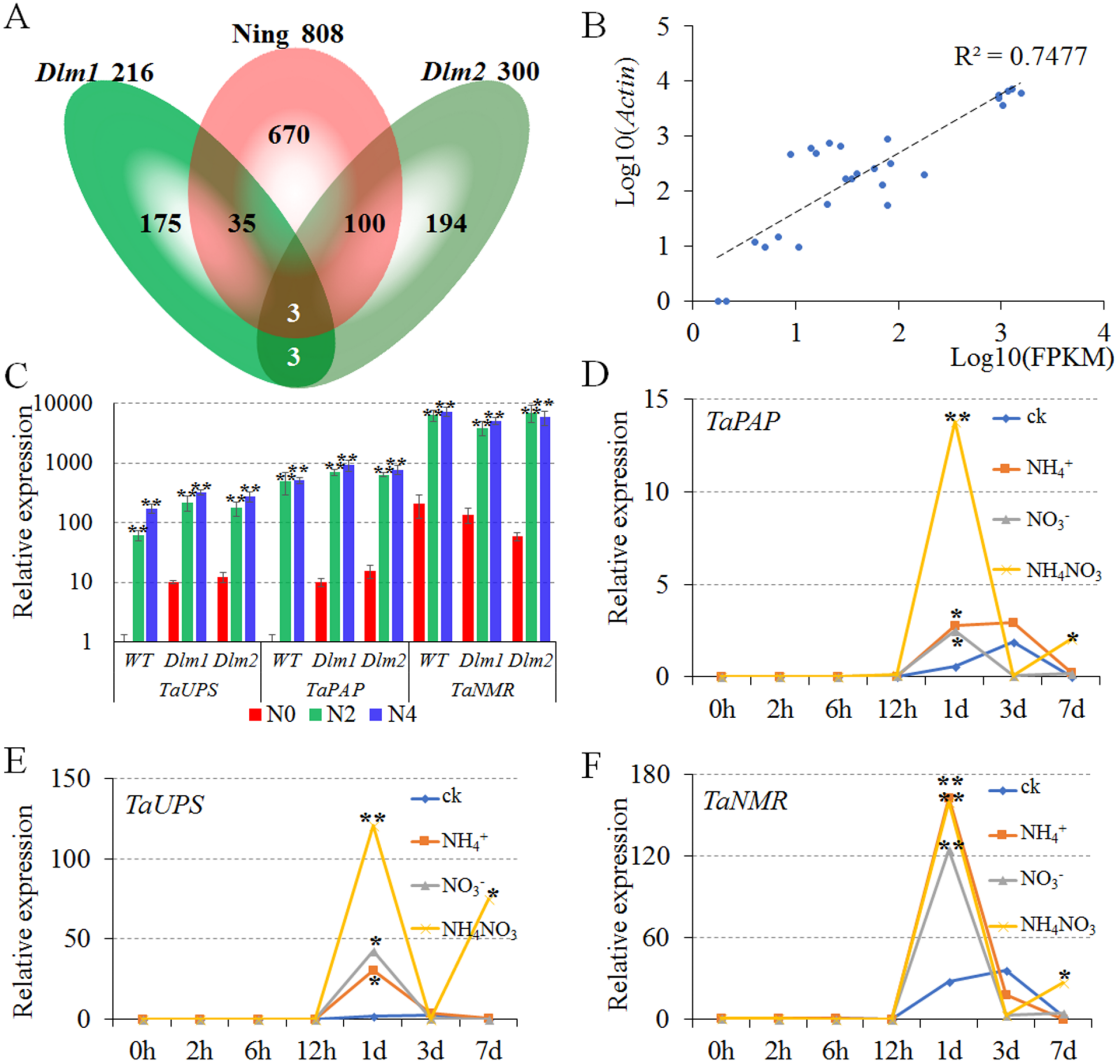
Verification and the expression patterns of conserved nitrogen metabolism-related genes. N0, N2 and N4 represent the treatment with 0 g, 2 g or 4 g of carbamide per pot, respectively. (A) Venn diagram of nitrogen-related DEGs unique and common with the number of differentially expressed genes. (B) The relative expression level of DEGs (Log10(*Actin*)) was highly correlated with the FPKM of transcriptome analysis (Log10(FPKM)). (C) The *TaPAP*, *TaUPS* and *TaNMR* were significantly up-regulated under N2 and N4 treatments compared to N0 in Ning7840, *Dlm1* and *Dlm2* mutants. (D–F) Expression pattern of *TaPAP* (D), *TaUPS* (E) and *TaNMR* (F), respectively. The expression of *TaPAP*, *TaUPS* and *TaNMR* were significantly up-regulated at 1 d under all the nitrogen treatments and were significantly up-regulated at 7 d only under the treatment with NH_4_NO_3_ when compared to CK.

Numbers of common DEGs of N0 vs. N2 and N0 vs. N4 were counted as 808, 216 and 300 in Ning7840, *Dlm1* and *Dlm2*, respectively. In order to find the conserved N-metabolism-related genes, we compared the common DEGs of N0 vs. N2 and N0 vs. N4 among Ning7840, *Dlm1* and *Dlm2*. The specific DEGs in Ning7840, *Dlm1* and *Dlm2* were 670, 175 and 194, respectively. Transcripts *comp106201_c0*, *comp107474_c0* and *comp95880_c0* were common DEGs across Ning7840, *Dlm1* and *Dlm2* mutants (Fig. 2A and Table S3). These three common DEGs were up-regulated under N2 and N4 compared to N0 in Ning7840, *Dlm1* and *Dlm2* mutants.

Transcripts annotation showed that: (1) *comp106201_c0* (hereafter abbreviated as *TaPAP*) encodes a purple acid phosphatase (PAP) belonging to MPP (metallo-phosphatase) super family; (2) *comp107474_c0* (hereafter abbreviated as *TaUPS*) encodes a ureide permease which has ten putative transmembrane domains with a large cytosolic central domain containing a Walker A motif; (3) *comp95880_c0* (hereafter abbreviated as *TaNMR*) has no putative conserved domains compared to known proteins by blasting against the NCBI database, and thus could be a novel nitrogen metabolism-related gene. The best hits of *TaPAP*, *TaUPS* and *TaNMR* were TraesCS3B02G359800, TraesCS7D02G466800 and TraesCS2A02G493400 respectively by blasting against the wheat RNAseq data. These genes were highly expressed in leaf tissue (http://www.wheat-expression.com).

### Verification of expressions of the three conserved N-metabolism- related genes

To validate the DEGs of transcriptome analysis, qPCR was performed for Ning7840, *Dlm1* and *Dlm2* mutants with *actin* as a reference gene at the heading stage under the same treatments in transcriptome analysis (three levels of N fertilizer). The results showed that the relative expression levels were highly correlated (*R*^2^ = 0.7477) with the FPKM of transcriptome analysis (Fig. 2B), and the *TaPAP*, *TaUPS* and *TaNMR* were significantly up-regulated under N2 and N4 compared to N0 in Ning7840, *Dlm1* and *Dlm2* mutants (Fig. 2C).

### Responses of the three conserved genes under different N-chemotype treatments

To validate the gene expression patterns of the three conserved N-metabolism-related genes under treatments with different chemotypes of N (NH_4^+^_, NO_3^−^_ and NH_4_NO_3_), qPCR was performed for Ning7840 with *actin* as a reference gene at the 5-leaf stage under treatments with three different N chemotypes. qPCR showed:

(1) Compared to CK, the expression of *TaPAP* had no significant difference among the three N-chemotype treatments within 12 h, however it was significantly increased at 1 d under all three treatments. The expression of *TaPAP* under NH_4_NO_3_ treatment was significantly higher than the other two N chemotypes and CK at 7 d (Fig. 2D).

(2) The expression patterns of *TaUPS* were similar to *TaPAP*, which had no significant difference among the three treatments within 12 h, but were significantly increased at 1 d under all treatments and down-regulated at 3 d. Compared to the CK, the expression of *TaUPS* was up-regulated at 7 d under NH_4_NO_3_ treatment only (Fig. 2E).

(3) No significant difference was detected in expression of *TaNMR* among the three treatments within 12 h, but the expression significantly increased at 1 d under all the treatments and down-regulated in the next six days. The expression of *TaNMR* under NH_4_NO_3_ treatment was significantly higher than the CK at 7 d, but was lower than that at 1 d (Fig. 2F).

In brief, the expressions of *TaPAP*, *TaUPS* and *TaNMR* were significantly up-regulated at 1 d under all three chemotype treatments, and were significantly up-regulated at 7 d only under NH_4_NO_3_ treatment when compared to the CK, indicating that *TaPAP*, *TaUPS* and *TaNMR* are involved in N metabolic activities.

## Discussion

### LM phenotype in Ning7840 is N-regulated

LM phenotypes in previous studies were either humidity and/or temperature regulated (Noutoshi et al., 2005; Sohn et al., 2014), light-induced (Arase et al., 2000; Ueno, Kihara & Arase, 2015; Yao et al., 2009) or day-length regulated (Ishikawa et al., 2001). Recently, we found that N regulates the LM phenotype in wheat variety P7001, and the LM disappeared under high dosages of N supply (Li et al., 2016a). In our previous study, we found that wheat variety Ning7840 has LM phenotype (Li & Bai, 2009; Li, Bai & Gu, 2012), and the current study showed that the LM phenotype in Ning7840 is also regulated by N. LM in other plants such as Arabidopsis, rice, maize and barley have also been reported, but the relationship between N supply and LM traits remains to be determined. P7001 and Ning7840 are unrelated to each other according to their pedigrees, suggesting N may play an important role in the regulation of LM expression in wheat.

### Identification of potentially conserved N-metabolism-related genes

N-metabolism is a complicated process which contains the uptake of nitrate, the reduction of nitrate and nitrite, the assimilation of ammonium and the catabolism of ammonium. Wheat variety Ning7840 has an N dependent LM phenotype, and its *Dlm1* and *Dlm2* mutants are non-LM. Our previous results showed that the proteasome pathway may be associated with the suppression of LM in *Dlm1* and the chlorophyll pathway is involved in the enhancement of photosynthesis and the suppression of LM in *Dlm2* mutant (Li et al., 2016b). Transcriptome analysis of wheat genotypes with visibly differential phenotypic responses to N supply could help uncover potentially conserved N-metabolism-related genes.

In Ning7840, 808 genes were common DEGs over N0 vs. N2 and N0 vs. N4. These DEGs are involved in metabolic pathways of amino acids such as alanine, aspartate, glutamate and phenylalanine, etc. In the *Dlm1* mutant, the glutathione metabolism pathway and the alanine, aspartate and glutamate metabolism pathways showed significant differences among different N levels treatments. The histidine metabolism pathway was up-regulated under treatments with high levels of N supply in the *Dlm2* mutant. Although many DEGs between the different N treatments have been found among Ning7840, *Dlm1* and *Dlm2*, only three genes were differentially expressed across the three genotypes over the three N levels, suggesting these three genes are probably conserved in N-metabolism and play a critically important role in regulation of N activities in wheat.

### The possible function of the three genes

N-metabolism genes such as NRTs, AMTs, NRs and NiRs are up-regulated rapidly after N treatment. The expression level of *CHL1* (classified as an NRT transporter) is rapidly up-regulated in 30 min and reaches a peak within 2 h. Some known N-metabolism genes were not detected in this study, which may be due to the immediate response of these genes under N treatment, whereas our transcriptome data was collected 7 days after N treatments. NRTs, AMTs, NRs and NiRs functioned in the uptake and transport of nitrate and ammonium or in the reduction of nitrate and nitrite. Our study showed that many differentially expressed genes are involved in the metabolism of amino acids such as glutamate, alanine and aspartate.

*TaPAP*, *TaUPS* and *TaNMR* were all up-regulated across the treatments with different N chemotypes when compared to the control. However, their expression patterns and intensities varied across N chemotypes. *TaUPS* and *TaNMR* showed an 8.6-fold intensity of *TaPAP* at 1 d under NH_4_NO_3_ treatment, and both *TaPAP* and *TaUPS* showed significantly higher expression intensities at 1 d under NH_4_NO_3_ treatment than their corresponding intensities at 1 f under NH_4^+^_ and NO_3^−^_ treatments, and up-regulations of *TaNMR* were not significant among the three N chemotypes. These observations suggested that *TaPAP* and *TaUPS* may be subjected to the interaction between NH_4_+ and NO_3_-chemotypes and *TaNMR* is not, and it is also possible that these three genes may be involved in distinct pathways of N-metabolism and may perform different functions.

In plants, most PAPs are unspecific, and phosphate-regulated PAPs can be secreted outside the root cells to the extracellular environment, hydrolyzing a broad spectrum of phosphate esters including ATP, PEP (phosphoenolpyruvate), sugar, phosphates, mononucleotides and inorganic pyrophosphate (Olczak, Morawiecka & Watorek, 2003). Many studies showed that N fertilization significantly increases the activities of acid phosphatase in the soil (Ajwa, Dell & Rice, 1999; Saiya-Cork, Sinsabaugh & Zak, 2002). Our study showed that addition of different chemotypes of N up-regulated the expression of *TaPAP* in leaf tissue of wheat.

UPS encoded a ureide permease which is involved in the transport of N compound. *AtUPS1* was the first reported member of UPS family that is involved in the uptake of allantoin and other purine degradation products such as uric acid and xanthine (Desimone et al., 2002). The up-regulation of *TaUPS* after N treatments in this study implied the response of *TaUPS* to N fertilization.

Sequence analysis of *TaNMR* showed that it has two homologous genes (*AK357114.1* and *AK355495.1*) in barley without known conserved domains. *AK357114.1* and *AK355495.1* are expressed in the shoot at the seedling stage (Matsumoto et al., 2011).

## Conclusions

*TaPAP*, *TaUPS* and *TaNMR* are the three conserved components responsive to all three different chemotypes of N across the three genotypes, and the former two have been proved to be involved in N-metabolism. We concluded that *TaNMR* is most likely an important new player associated with N-metabolism in wheat.

##  Supplemental Information

10.7717/peerj.7281/supp-1 Figure S1 The size distribution of 65,383 transcripts A total of 65,383 transcripts were obtained from the nine assembled samples. The N50 (the length for which the sum of bases in the long contig of that length or longer is at least half the bases in the assembly) of transcripts is 1,425 nt.

10.7717/peerj.7281/supp-2 Table S1 primer sequence and statistics of clean reads (A) Oligonucleotide primers used for qPCR. (B) Statistics of clean reads.

10.7717/peerj.7281/supp-3 Table S2 The gene expression levels in Ning7840 and the *dlm* mutants

10.7717/peerj.7281/supp-4 Table S3 Annotation and sequence of the three nitrogen metabolism-related genes

10.7717/peerj.7281/supp-5 File S1 Sequence of the three nitrogen metabolism related genes

## Additional Information and Declarations

### Data Availability

The following information was supplied regarding data availability: Data is available at NCBI under accession number PRJNA526019. The sequences of conserved N-metabolism-related genes are available as Supplemental File.
