# Supplementary figures and images for: Identification of conserved genes involved in nitrogen metabolic activities in wheat
Source: PeerJ. 2019 Jul 9;7:e7281. doi: 10.7717/peerj.7281 (PMC6625498; doi:10.7717/peerj.7281)

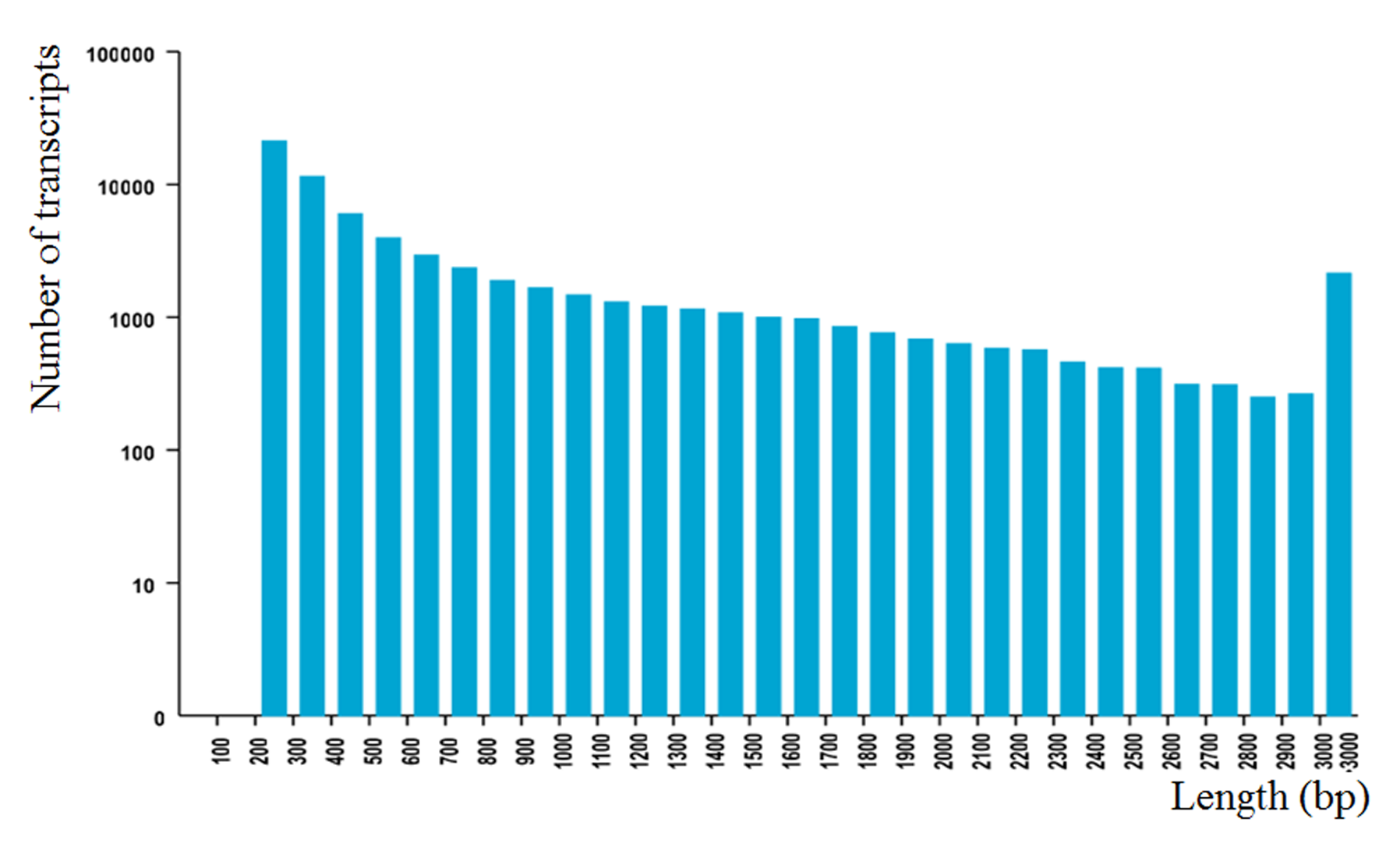

Supplement: Figure S1 — A total of 65,383 transcripts were obtained from the nine assembled samples. The N50 (the length for which the sum of bases in the long contig of that length or longer is at least half the bases in the assembly) of transcripts is 1,425 nt. [file peerj-07-7281-s001.png]
